# Is it safe for women with a history of two cesarean deliveries to undergo a vaginal delivery attempt in comparison to patient with a history of one cesarean delivery?

**DOI:** 10.1371/journal.pone.0330216

**Published:** 2025-08-14

**Authors:** Anna Elisabeth Hentrich, Dörthe Brüggmann, Samira Catharina Hoock, Eileen Deuster, Jakob Thomas Buslowicz, Juliane Bresgen, Nadja Zander, Frank Louwen, Lukas Jennewein

**Affiliations:** 1 Department of Obstetrics and Perinatal Medicine, University Hospital, Goethe University Frankfurt, Frankfurt, Germany; 2 Department of Anaesthesiology, Intensive Care Medicine and Pain Therapy, University Hospital, Goethe University Frankfurt, Frankfurt, Germany; Xiangya Hospital Central South University, CHINA

## Abstract

**Introduction:**

Worldwide, numbers of repeat cesarean sections continue to rise. Although there is a multitude of evidence about the safety of a vaginal delivery attempt after one cesarean section, data is scarce regarding the risks of one compared to two prior procedures. This study aims to determine whether vaginal childbirth is less safe and successful for both mother and child in patients with a history of two cesarean sections compared to those with only one.

**Materials and methods:**

This retrospective cohort study included all patients with a history of one or two prior cesarean deliveries who gave birth at term at Goethe University Frankfurt between 2014 and 2021. Maternal and neonatal morbidity, as well as rates of success for vaginal birth and uterine rupture, were compared between the groups.

**Results:**

Of the 1967 women studied, 1697 gave birth after one previous cesarean section, while 270 had a history of two prior cesarean sections. There was no significant increase in maternal or fetal morbidity in women with two previous cesarean sections compared to one. However, the success rate of a vaginal delivery was lower in the group with two prior cesareans (27/79, 34.2%) than in the group with one (696/989, 70.3%). The rate of complete uterine rupture was higher in patients with two cesareans who underwent cesarean section after onset of labor (CSAOL-2: 3/89, 3.4%) compared to none in the CSAOL-1 group (0/492, 0.0%; p = 0.004). Maternal and fetal morbidity remained comparable across groups, with NICU admission rates of 11.2% (CSAOL-1) vs. 5.6% (CSAOL-2), and maternal transfusion rates of 0.86% (VBAC-1) vs. 6.45% (VBAC-2).

**Conclusion:**

In the context of patient autonomy regarding the timing of delivery, offering the patient the choice of different delivery modes after two previous cesareans appears to be safe with respect to maternal and fetal risks. An individualized consultation and thorough counseling are essential, but the opportunity for different delivery options should be respected and supported.

## Introduction

Over the last decades the number of planned repeat cesarean sections has continued to rise. In 1996, 30.2% of patients with a history of cesarean section opted for a vaginal delivery, while only 11.3% chose this option in 2003 [1]. Following the 2010 update of the ACOG guidelines, the repeat cesarean section rate in the US reached 85.9% [1]. This development has contributed an increase in the number of repeat cesarean sections performed worldwide, leading to short- and long-term morbidities associated with this clinical practice. Numerous national and international guidelines – e.g., issued by ACOG [2,3], RCOG [4] and DGGG [3] – recommend counselling women about the safe option of a vaginal birth after cesarean section (VBAC). Nevertheless, many patients still consider a planned cesarean delivery to be a reasonable choice. Intentions regarding this decision are often encouraged by medical providers and include better scheduling, fear of uterine rupture, medico-legal considerations, lack of medical experience or psychological reasons.

Clinical evidence supporting the safety of vaginal birth after one cesarean section is solid. In multiple randomized studies no significant differences were detected in maternal and fetal morbidity rates when comparing VBAC and elective cesarean sections [5]. Hence, a first cesarean section (if performed with a low vertical incision) should never be the sole indication for an elective repeat procedure. Guidelines recommend that only a history of *three* cesarean sections is a definitive indication for another repeat surgical delivery. As a consequence, healthcare providers play a crucial role conselling patients, who are seeking a vaginal delivery after *two* cesarean sections. In the context of self-determination/patient autonomy and predictability [6], it is understandable that women – particularly those who desiring further pregnancies – want to avoid additional surgeries associated with risks such as hemorrhage, embolism and placenta accreta spectrum.

Further evidence is needed to identify which patients are suitable to safely undergo a desired trial of labor after *two* cesarean sections. The aim of this study is to investigate whether the delivery process, specially the onset of labor, is less safe for both mother and child in patients with two previous cesarean sections compared to those with just one. Specifically, we aimed to compare [1] the maternal and fetal morbidity associated with different birth modes after the onset of labor from late preterm to term gestation [2], the success of an intended vaginal birth and [3] the uterine rupture rate in patients with a history of *two* versus *one* prior cesarean section.

## Materials and methods

### Patient cohort and collective

In this retrospective cohort analysis, we included all patients from our data base with deliveries at 36 0/7–41 6/7 weeks’ gestation and a history of one or two previous cesarean sections. The participants gave birth in the Department of Obstetrics and Perinatal Medicine at the Goethe University Hospital Frankfurt (a center providing highest level of perinatal care in Hesse, Germany) between 01/01/2014 to 30/062021.

The authors obtained study approval from the local research ethics committee (Internal Reference Number: #2021-325). A specific informed consent of the patients was waived since the outcome variables were generated during routine patient care and collected in retrospective fashion. All patient data were analyzed anonymously with strict privacy policies in place from 01/06/2022–31/12/2023.

Inclusion criteria were 36 0/7–41 6/7 weeks’ gestation, maternal age > 18 years and under 45 years, live fetus and a history of one or two cesarean sections.

**Fig 1:**
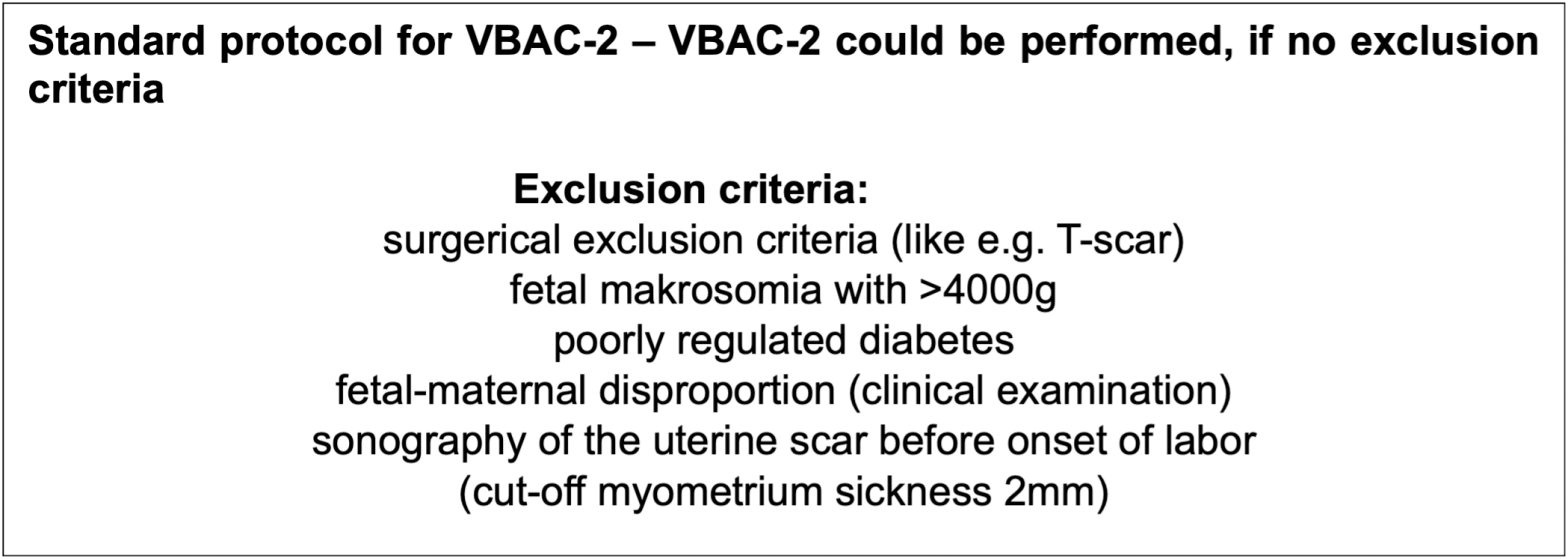
Standard protocol with exclusion criteria.

Exclusion criteria included major fetal malformations, contraindications for vaginal delivery (e.g., an uterine T-scar and myoma enucleation) and delivery before 36 0/7 weeks of gestation and twin pregnancy. Patients were included with a defined standardized protocol (Fig 1), so if they do not implement these criteria, they were excluded from VBAC-2.

The exclusion criteria were established during the consultation for delivery mode planning (between the 36th and 38th week of pregnancy). Fetal macrosomia was defined as an estimated weight above the 90th percentile or a birth weight of previous children over 4000g, with the expectation that the current child would fall into this range. Poorly controlled diabetes mellitus was defined as more than three fasting or more than three postpartum outliers in one week, or sonographic criteria such as polyhydramnios or a head-abdomen discrepancy. Indicators of fetomaternal disproportion were primarily provided by the medical history (obstetric arrest during the expulsion phase in both previous cesareans) as well as clinical examination (relation of the head to the pelvis at the onset of labor). The measurement of the lower uterine segment was performed as described by Kok et al [7].

### Data collection

Patient medical records (both paper and electronic) and nationwide delivery database (“Perinatalerhebung Hessen”) were utilized for patient identification and outcome data collection. In addition to the intended mode of delivery, we evaluated the actual mode of birth. Patients were divided into the following groups:

1. **Vaginal birth after cesarean section (VBAC)**: after one (VBAC-1) or two previous cesarean deliveries (VBAC-2), including spontaneous vaginal births and those assisted by forceps.
2. **Cesarean section after onset of labor (CSAOL)**: after one (**CSAOL**-1) or two cesarean deliveries (CSAOL-2).

The CSAOL group included patients who opted for a planned repeat cesarean and experienced contractions before the elective delivery date as well as patients, who desired a cesarean section after the onset of contractions or a rupture of membranes.

3 **failed Trial of labor after cesarean section**: A subgroup of patients who failed the trail of labor and underwent a subsequent cesarean section (f**TOLAC**-1 after one and fTOLAC-2 after cesarean deliveries). Here, indications for cesarean sections included arrest in labor, imminent rupture (clinical symptomatic with pain in the scar area), non-reassuring fetal heart tone pattern and maternal wish.

The patients were cared for by a certified midwife, residents, and attending attending physicians, who are board-certified in Obstetrics and Gynecology.

### Outcome variables

**Primary outcomes** were maternal and fetal morbidity and mortality related to the actual mode of delivery and the patient’s history of one or two cesarean sections. Relevant **maternal morbidity** included operative complications like wound healing disorders, urinary bladder injury, blood loss (in ml), transfusion of blood products, uterine atony, hysterectomy, and higher-grade birth injuries. Higher-grade birth injuries were defined as third- or fourth-degree perineal tears and/or extensive vaginal or cervical lacerations requiring surgical repair.

**Fetal morbidity** included admission and stay (in days) in the Neonatal Intensive Care Unit (NICU), age (in days) at demission, APGAR <5 after 5 min (after birth), pH in the umbilical artery after birth, short-term problems with breathing as well as respiratory distress syndrome and neonatal infection.

Special interest was to compare the maternal and fetal outcome after vaginal birth after cesarean section (VBAC) as well as cesarean section after onset of labor (CSAOL) between patients after one cesarean section and after two cesarean sections to make clear if the maternal risks alter dependent on numbers of prior CS after the onset of labor.

**Secondary outcomes** were the success rates of vaginal birth after one and after two cesarean sections as well as the rates of uterine rupture. The occurrence of the outcome “uterine rupture” was extracted from surgery notes. Also, birth documentation was assessed whether the women had pain in/adjacent to the scar or a non-reassuring fetal heart tone tracing.

A ‘complete uterine rupture’ was defined as a full-thickness disruption of the uterine wall, including the serosa (as identified intraoperatively). A ‘covered uterine rupture’ was defined as a disruption of the myometrial layer that was stabilized by adjacent structures such as the bladder or peritoneum. Due to the retrospective design and limitations of operative documentation, covered ruptures were considered equivalent to uterine dehiscence in our analysis.

### Statistical analysis

Statistical analysis was performed using JMP 14.0 software (SAS Institute, Cary, NC, USA). Groups of variables were tested for normal distribution with Kolmogorov-Smirnov-Testing. Group differences of normal distributed values were tested with student’s t-test. Group differences of non-parametric variables were tested using Pearson’s χ^2^ test. A *p*-value of below 0.05 was considered as statistically significant.

## Results

**Fig 2:**
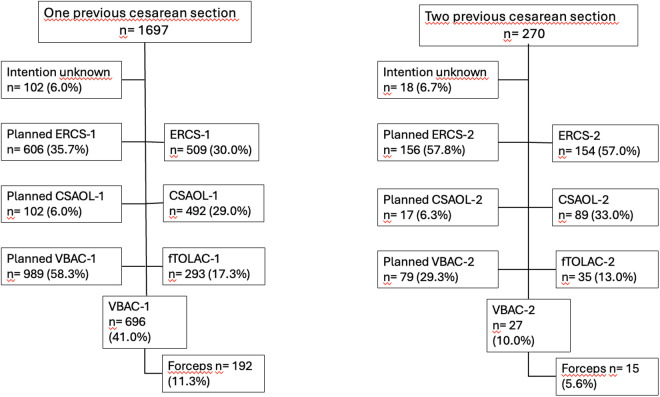
Chart of different delivery modes with one or two previous cesarean sections and the intended mode of delivery before labor onset. ERCS = elective repeat cesarean section, CSAOL = cesarean section after onset of labor, fTOLAC = failed trial of labor after cesarean, VBAC = vaginal birth after cesarean.

A total of 1967 women who had a history of one (n = 1697) or two (n = 270) previous cesarean sections were included in the study (Fig 2).

### Intended mode of delivery

After one prior cesarean section, 989 patients (58.3%) intended a vaginal delivery after cesarean section (VBAC-1), 102 patients (6.0%) desired a cesarean section after the onset of labor (CSAOL-1) and 606 women (35.7%) intended an elective planned repeat cesarean section before the onset of labor (ERCS-1). In 102 women (6.0%), information reading their intended mode of delivery could not be extracted from the records.

Out of 270 women, who had two cesarean sections in the past, 79 patients (29.3%) underwent a trial of labor after two cesarean sections. This particular mode of delivery required spontaneous onset of labor since a medical induction of labor would be contraindicated in our clinical standard protocol. Furthermore, 17 patients (6.3%) opted for a cesarean section after onset of labor (CSAOL-2), and n = 156 (57.8%) for an elective repeat cesarean section (ERCS-2). In 18 patients (6.7%), the intended mode of delivery was unknown.

### Actual mode of delivery

696 patients (70.3%) gave vaginal birth after **one prior cesarean** section (VBAC-1); 192 (27.6%) of these had a delivery assisted by forceps. Repeat cesarean sections after onset of labor and one previous cesarean section (CSAOL-1) were performed in 492 women (29.0%). Among these, 293 patients (59.6%) underwent a cesarean section after a failed trail of labor (fTOLAC-1).

A total of 27 patients (34.2%), including 4 with a history of vaginal birth, who had two previous cesarean sections, underwent a vaginal delivery (VBAC-2). Of these, 15 patients (55.6%) required forceps assistance.

Additionally, 89 patients (33.0%) underwent a repeat cesarean section after the onset of labor (CSAOL-2). In 52 of these cases (58.4%), the trial of labor failed, and a cesarean section was performed (fTOLAC-2).

### Characteristics of the study population

There were significant differences documented when maternal demographic data (such as BMI, age, etc) were compared between patient groups with one versus two cesarean sections (Table 1). Patients expecting their third child were on average older than patients giving birth to their second child (33.61 versus 38.57 years, p-value: 0.001).

**Table 1:** Characteristics of the study population.

Characteristics	Total 1CS n = 1697	Total 2CS n = 270	p-value
**Age (years, mean ±Standard Deviation SD)**	33.61 ± 4.76	38.57 ± 5.33	<0.001
**BMI (kg/m², mean ± SD)**	25.33 ± 5.49	27 ± 5.90	<0.001
**Parity**	2.42	3	<0.001
**Duration of pregnancy (weeks, mean ± SD)**	39.24 ± 1.13	38.75 ± 1.4	<0.001
**Gestational diabetes (n, %)**	231 (13.6)	44 (16.3)	0.256
**Preeclampsia (n, %)**	19 (1.19)	2 (0.74)	0.758
**Maternal coagulopathy (n, %)**	73 (4.30)	15 (5.56)	0.343
**Vaginal birth in history (n, %)**	231 (13.6)	25 (9.25)	0.032
**Fetal birth weight (grams, mean ± SD)**	3468.5 ± 485.6	3462.6 ± 494.37	0.779

When comparing fetal characteristics between the two groups, no statistically significant differences were observed in the majority of demographic data, such as weight. In both groups, just 13.6% women had a history of one previous cesarean, and 9.25% of two previous cesarean (p = 0.032). Out of them, just 7 patients (7/25) intended VBAC-2. So, 72 patients intended VBAC-2 without a history of vaginal birth (Table 1).

#### Primary outcome.

In this study, no maternal or fetal mortality was observed.

#### Maternal morbidity—VBAC.

The analysis of maternal and fetal morbidity in patients after VBAC revealed no significant differences between patients with one versus two prior CS, with the exception of peripartal blood transfusions. 6.45% of women after VBAC-2 required transfusion compared to 0.86% of VBAC-1 patients (p = 0.042, Table 2). In the VBAC-2 group, both cases (2/31, 6.45%), requiring blood transfusion occurred after operative vaginal delivery with Kielland forceps and were due to postpartum hemorrhage from vaginal lacerations. Episiotomy is not routinely performed at our institution, with an overall rate of only 1.8%, and neither of these cases involved an episiotomy.

**Table 2:** Maternal morbidity VBAC-1 vs. VBAC −2.

Maternal morbidity	VBAC 1 n = 696	VBAC2 n = 31	p-value
**Bladder injury (n, %)**	0	0	0
**Atonia (n, %)**	12 (1.72)	0	1.00
**Blood transfusion (n, %)**	6 (0.86)	2 (6.45)	0.042
**Hysterectomy**	0	0	
**Blood loss** **(ml, mean ± sd)**	340.26 ± 249.55	372.58 ± 237.28	0.077

#### Maternal morbidity—CSAOL and fTOLAC.

Patients undergoing a cesarean section after onset of labor (CSAOL) experienced no significant differences in complications translating in short-term morbidity (such as bladder injuries, uterine atony, blood loss or transfusion) when groups after one or two previous cesarean sections were compared (Table 3).

**Table 3:** Maternal morbidity CSAOL 1 vs CSAOL 2.

Maternal morbidity	CSAOL-1 n = 492	CSAOL-2 n = 89	p-value
**Wound infection (n, %)**	2 (0.41)	1 (1.12)	0.393
**Bladder injury (n, %)**	5 (1.01)	1 (1.12)	1.00
**Atonia (n, %)**	9 (1.83)	1 (1.12)	1.00
**Blood transfusion (n, %)**	2 (0.41)	0	1.00
**Hysterectomy**	0	0	
**Blood loss** **(ml, mean ± sd)**	451.86 ± 256.88	474.16 ± 219.78	0.052

The same finding – no significant differences in short-term morbidity after one or two cesarean sections – were documented in the subgroup of failed vaginal births and consecutive cesarean section (fTOLAC). e.g., there were just 3 patients with postpartum hemorrhage after one cesarean section and one patient after two cesarean sections (p-value 0.365, Table 4).

**Table 4:** Maternal morbidity fTOLAC-1 and fTOLAC −2.

Maternal morbidity	fTOLAC 1 n = 293	fTOLAC2 n = 35	p-value
**Bladder injury (n, %)**	3 (1.02)	0	1.00
**Atonia (n, %)**	3 (1.02)	1 (2.86)	0.365
**Blood transfusion (n, %)**	0	0	0
**Hysterectomy**	0	0	
**Blood loss** **(ml, mean ± sd)**	437.35 ± 176.60	514.29 ± 304.76	0.133

#### Fetal morbidity—VBAC.

Analysis of fetal morbidity after vaginal birth after cesarean section (VBAC) revealed a significant difference in the proportion of neonates with a base excess < −8 between groups: In the VBAC-1 group, 22.25% (154/696) of neonates had a base excess < −8, compared to only 6.45% (2/31) in the VBAC-2 group (p = 0.042, Table 4), indicating a higher rate of metabolic acidosis in the VBAC-1 group. In contrast, there were no significant differences between the groups in mean umbilical artery pH (VBAC-1: 7.21 ± 0.08; VBAC-2: 7.21 ± 0.05; p = 0.866) or in the proportion of neonates with pH < 7.15 (15.32% vs. 9.68%; p = 0.606). Thus, the significant difference was limited to the metabolic component (base excess), not the pH value. Fetal morbidity after VBAC was similar between the two groups, with no significant difference in the NICU admission rates or other fetal outcomes (Table 5).

**Table 5:** Fetal morbidity VBAC-1 vs. VBAC −2.

Fetal Morbidity	VBAC 1 n = 696	VBAC2 n = 31	p- value
**pHa (mean± SD)**	7.21 ± 0.08	7.21 ± 0.05	0.866
**pHa < 7,15 (n, %)**	106 (15.32)	3 (9.68)	0.606
**Base Excess < −8 (n, %)**	154 (22.25)	2 (6.45)	0.042
**APGAR 5’ < 4 (n, %)**	4 (0.58)	0	1.00
**Intubation (n, %)**	2 (0.29)	0	0.497
**Admission to NICU (n, %)**	38 (5.46)	1 (3.33)	1.00
**Short term breathing problems (n, %)**	13 (1.87)	0	1.00
**Perinatal asphyxia (n, %)**	3 (0.43)	0	1.00
**Respiratory distress (n, %)**	1 (0.14)	0	1.00
**Amnioninfection (n, %)**	8 (1.15)	0	1.00
**Fetal death**	0	0	

#### Fetal morbidity—CSAOL and fTOLAC.

The fetal outcome showed no significant differences in the situation of a cesarean section after the onset of labor (CSAOL, Table 6).

**Table 6:** Fetal morbidity CSAOL-1 vs. CSAOL-2.

Fetal Morbidity	CSAOL-1 n = 492	CSAOL-2 n = 89	p- value
**pHa (mean± SD)**	7.30 ± 0.068	7.30 ± 0.056	0.656
**pHa < 7,15 (n, %)**	19 (3.86)	0	0.096
**Base Excess < −8 (n, %)**	3 (3.37)	17 (3.46)	1.00
**APGAR 5’ < 4 (n, %)**	1 (0.20)	0	1.00
**Intubation (n, %)**	7 (1.42)		0.602
**Admission to NICU (n, %)**	55 (11.18)	5 (5.62)	0.131
**Days at NICU (n, %)**	1.05 (5.59)	0.35 (1.64)	0.112
**Short term breathing problems (n, %)**	28 (5.68)	1 (1.12)	0.106
**Perinatal asphyxia (n, %)**	3 (0.61)	0	1.00
**Respiratory distress (n, %)**	6 (1.22)	0	0.598
**Amnioninfection (n, %)**	16 (3.25)	0	0.149
**Fetal death**	0	0	

When fTOLAC groups after one or two previous cesarean sections were compared the fetal outcome did not differ between the two groups (Table 7).

**Table 7:** Fetal morbidity fTOLAC-1 and fTOLAC −2.

Fetal Morbidity	fTOLAC1 n = 293	fTOLAC2 n = 35	p- value
**pHa (mean± SD)**	7.29 ± 0.07	7.3 ± 0.07	0.214
**pHa < 7,15 (n, %)**	15 (5.12)	0	0.384
**Base Excess < −8 (n, %)**	14 (4.78)	2 (5.71)	0.684
**APGAR 5’ < 4 (n, %)**	1 (0.34)	0	1.00
**Intubation (n, %)**	6 (2.05)	0	1.00
**Admission to NICU (n, %)**	35 (11.95)	1 (2.86)	0.15
**Short term breathing problems (n, %)**	20 (6.83)	1 (2.86)	0.712
**Perinatal asphyxia (n, %)**	3 (1.02)	0	1.00
**Respiratory distress (n, %)**	5 (1.71)	0	1.00
**Amnioninfection (n, %)**	13 (4.44)	0	0.375
**Fetal death**	0	0	

#### Secondary outcome.

The **success rate** of a vaginal delivery was twice as high as in VBAC-1 (70.3%) compared to VBAC-2 patients (34.2%) (Table 8).

**Table 8:** Success rate VBAC-1 vs. VBAC-2.

	VBAC-1	VBAC-2
**Intented VBAC n**	989	79
**Success (n, %)**	696 (70.30%)	27 (34.17%)
**failed TOLAC (n, %)**	293 (29.62%)	52 (65.82%)

Complete uterine rupture occurred in 3 out of 89 patients (3.37%) in the CSAOL-2 group (after two cesarean sections), while no cases were observed in the CSAOL-1 group (after one cesarean section) All three cases of complete uterine rupture in the CSAOL-2 group occurred in women with two previous cesarean sections. In all three cases, the patients presented with the onset of irregular contractions, still in the latent phase of labor. None of these women had intended a trial of labor after two cesarean sections (VBAC-2); all had planned for a repeat cesarean section. The diagnosis of uterine rupture was made after the onset of pain and prior to active labor.

When CSAOL groups were compared, the total rate of uterine ruptures was not significantly higher in the group of patients after one compared to two cesarean sections in the past (difference of n = 48 9.8% versus n = 14 15.7%, p = 0.095, Table 9).

**Table 9:** Rupture rate in patient group CSAOL.

	CSAOL-1 n = 492		CSAOL-2 n = 89		p-value
**Complete rupture (n, %)**	0	(0)	3	(3.37)	0.004
**Total rupture rate (n, %)**	48	(9.76)	14	(15.73)	0.095

No cases of complete uterine rupture occurred in women who underwent a failed trial of labor after cesarean section (fTOLAC) and subsequently required a cesarean delivery—neither in the group with one prior cesarean section nor in the group with two prior cesarean sections. (Table 10). The total rate of uterine ruptures did not differ significantly between both fTOLAC groups.

**Table 10:** Rupture rate in patient group fTOLAC.

	fTOLAC-1 n = 293	%	fTOLAC-2 n = 35	%2	p-value
Complete rupture	0		0		
Total rupture rate	33	11.3	4	11.4	1.00

Among the 989 women who intended a vaginal birth after one prior cesarean section (VBAC-1), labor was induced in 110 cases and occurred spontaneously in 879 cases. Uterine rupture was observed in 11 of the induced cases (10.0%) and in 47 of the spontaneous cases (5.27%), with a p-value of 0.052.

In both groups, all ruptures were classified as covered uterine ruptures (muscular dehiscence). No complete (open) uterine ruptures were documented in either group.

## Discussion

This study showed no relevant increase in maternal and fetal risks due to the “onset of labor” in deliveries of patients after one compared to those after two cesarean sections.

Overall, our data did not reveal significant differences in maternal or fetal morbidity between women with one versus two previous cesarean sections undergoing CSAOL or fTOLAC. However, an important exception was observed in the VBAC groups: the need for maternal blood transfusion was significantly higher in the VBAC-2 group (6.45%, 2/31) compared to the VBAC-1 group (0.86%, p = 0.042). Upon closer analysis, this increased transfusion rate was exclusively associated with two women in the VBAC-2 group who underwent operative vaginal delivery by Kielland forceps and experienced bleeding from vaginal lacerations. Apart from this specific finding related to operative vaginal births in the VBAC-2 group, we did not observe an overall increase in other maternal morbidity risks.

Our data are thus consistent with findings of two large investigations, a large prospective multicenter observational study authored by Landon et al. as well as a retrospective cohort analysis by Macones et al. [8,9]In contrast, other literature can be identified which described an increase in the maternal complication rate due to the onset of labor [10,11] These discrepancies may be attributed to differences in provider experience, patient management strategies during labor—such as the use of oxytocin augmentation as a risk factor for atony—or to differences in the detailed capture of specific complications in our study. Some reviews discuss the increasing fetal risks after “onset of labor” [12–14], even though the absolute fetal risk increase has never been shown [10,15] A systematic review by Tahseen et al. also shows no changes in neonatal outcomes when comparing VBAC-1 vs. VBAC-2 [16].

The patients, who intended a vaginal birth in this study, experienced a vaginal delivery in 70% after one and 34% after two cesarean sections. of these, 27.5% and 55.5% respectively were delivered by forceps. At 70%, our VBAC-1 rate is in the lower segment within the success rates reported in the literature [8,9,13]. Overall, the **success rate** of a vaginal delivery was twice as high as in VBAC-1 compared to VBAC-2 patients. The success rate of VBAC-2 is clearly lower in our study than the reported evidence [8,16]. Reasons for the low success rate may include suboptimal patient selection despite the applied standard protocol, particularly in relation to fetomaternal disproportion. Certainly, the individual clinical situation and the experience of the attending physicians will also play a role. This may also explain the increased rate of vaginal operative deliveries. An increased rate of vaginal operative deliveries was also observed by Denjean et al. [17]. The study comes to the same conclusion as our data: a vaginal birth after two cesarean sections should be offered to a patient, but counseling about the increased risk of a vaginal operative delivery must be provided. In our study, it was evident that the need for maternal blood transfusions increased in successful vaginal births (especially vaginal-operative birth) of patients after two versus one prior cesarean section. This finding is confirmed by other evidence [8,10,17].

However, the delivery notes listed “birth injuries” as the cause for maternal blood loss in our study – not uterine atony. In other studies the reason for the blood transfusion is not reported [10].

Regarding the **uterine rupture rate**, there is no significant difference between women who had a fTOLAC-1 versus fTOLAC-2. The occurrence of complete uterine rupture exclusively in the CSAOL-2 group (3/89, 3.37%) and not in the CSAOL-1 group is consistent with the established literature showing an increased risk of uterine rupture with a higher number of previous cesarean sections. Upon detailed review, all three cases in our cohort occurred in women who had not intended a trial of labor after two cesarean sections and who presented with irregular contractions in the latent phase, with cervical dilation not exceeding 1–2 cm. Importantly, in all three cases, the indication for emergency cesarean section was a pathological fetal heart rate tracing (CTG), and the diagnosis of uterine rupture was made after the onset of pain and prior to active labor. This finding is consistent with the literature demonstrating an increased risk of uterine rupture with a higher number of previous cesarean sections, even though the absolute risk remains low. Our data highlight that complete uterine ruptures may occur even before the onset of active labor and in the absence of a planned trial of labor, emphasizing the need for close monitoring in women with two prior cesarean deliveries, especially when symptoms such as pain or abnormal fetal heart rate patterns arise. Nevertheless, we want to underline the fact that even if the complete rupture rate is significantly higher in CSAOL-2, it did not translate into differences regarding the maternal or fetal outcomes [8]. There are only a few studies published about the safety of a trial of labor after multiple cesareans [18]. In numerous prospective studies, the authors did also not report an increase in uterine rupture rates [8,19]. Especially, Zwergel et al. showed that the rupture rate increases significantly only after 3 prior cesarean sections [18,20]. However, the overall rupture rate is not significantly increased in our study due an onset of labor. This is consistent with the data in the literature that although the uterine rupture risk is increasing with the number of cesarean sections in the patient’s past, the absolute risk is still low after two cesareans [18]. Overall, based on our institutional experience, most complete uterine ruptures observed at our center between 2014 and 2021 occurred in cases that would have been excluded from this study, such as preterm deliveries or women with a history of myoma enucleation. The WHO Analysis of the issue “Uterine Rupture” reports similar findings [21].

In our study, the status of having one previous CS was not associated with an increased risk of uterine rupture. This result aligns with the WHO Analysis and other studies [21,22]. Even the differences in the duration of pregnancy between our two patient groups are to explain, because of the possibility of induction of labor after due date after one cesarean section in our standard protocoll. Miller et al. demonstrate in their study the safety of labor induction even after two cesarean sections [23]. However, our evaluation is based on a retrospective design, which adhered to the clinic’s standard protocol. Reconsidering our protocol in light of the data, however, is open for discussion.

### Strength and weaknesses

Although only a small number of women with a history of two previous cesarean sections achieved a vaginal birth in our study (n = 27), only 4 of these women had experienced a previous vaginal delivery. This low proportion of women with prior vaginal birth among successful VBAC-2 cases is noteworthy, as previous vaginal delivery is a well-established predictor of VBAC success. Our findings therefore suggest that, even in the absence of a prior vaginal birth, selected women with two previous cesarean sections may still achieve a successful vaginal delivery. This distinguishes our cohort from other studies, where a higher proportion of successful VBAC-2 cases often had a history of prior vaginal birth. A weakness of the study are the retrospective design and the small sample size. Despite the existing standard protocol, the low success rate of VBAC-2 should be discussed. Although our data show that fTOLAC does not present measurable disadvantages for the patients, the personal disappointment of the patients must still be taken into consideration.

Due to the retrospective design of our study and the limitations of intraoperative documentation, a strict differentiation between covered uterine rupture and muscular dehiscence was not always possible. We made every effort to separate these entities based on the available records, but some overlap cannot be excluded. This may have contributed to higher reported rates of uterine rupture compared to studies with stricter prospective definitions. Readers should consider this limitation when interpreting our findings.

## Conclusion

Our study demonstrates that, with careful patient selection and thorough counseling, a trial of labor at term after two previous cesarean sections can be considered a reasonable option. Most maternal and fetal outcomes did not differ significantly between women with one versus two prior cesarean sections. However, we observed a higher rate of blood transfusion following operative vaginal delivery and a higher absolute rate of uterine rupture in women with two prior cesarean sections, although the total number of events was small.

Importantly, our data also show that women with two previous cesarean sections had a significantly lower success rate for vaginal birth and a notably higher rate of instrumental vaginal deliveries compared to women with only one prior cesarean section. These findings highlight the increased risk of unsuccessful vaginal birth and the greater likelihood of requiring operative assistance in this group.

Taken together, these results underscore the need for individualized risk assessment, close intrapartum monitoring, and transparent counseling for women considering a trial of labor after two cesarean sections. While the option for different delivery modes should be respected and supported, patients must be informed about the specific risks—including the higher likelihood of failed vaginal birth and instrumental delivery—associated with their obstetric history.

## Abbreviations

VBAC: vaginal birth after cesarean section
VBAC-1: vaginal birth after one cesarean section
VBAC-2: vaginal birth after two cesarean section
CSAOL: cesarean section after onset of labor
CSAOL1: cesarean section after onset of labor after one previous cesarean section
CSAOL2: cesarean section after onset of labor after two previous cesarean section
fTOLAC: failed trial of labor after cesarean section
fTOLAC1: failed trial of labor after one previous cesarean section
fTOLAC2: failed trial of labor after two previous cesarean section
CS: cesarean section
ERCS: elective repeat cesarean section
ERCS 1: elective repeat cesarean section after one previous cesarean section
ERCS 2: elective repeat cesarean section after two previous cesarean section.
